# An Evaluation of Neoadjuvant Chemoradiotherapy for Patients with Resectable Pancreatic Ductal Adenocarcinoma

**DOI:** 10.1155/2013/298726

**Published:** 2013-06-20

**Authors:** Hui Jiang, Chi Du, Mingwei Cai, Hai He, Cheng Chen, Jianguo Qiu, Hong Wu

**Affiliations:** ^1^Department of Hepatobiliary Pancreatic Surgery, The Second People's Hospital of Neijiang, Luzhou Medical College, Neijiang, Sichuan 641003, China; ^2^Department of Cancer, The Second People's Hospital of Neijiang, Luzhou Medical College, Neijiang, Sichuan 641003, China; ^3^Department of Hepatobiliary Pancreatic Surgery, West China Hospital, Sichuan University, Chengdu, Sichuan 610041, China

## Abstract

*Aims.* The aim of this study is to compare our results of preoperative chemotherapy followed by pancreaticoduodenectomy (PD) with those of surgery alone in patients with localized resectable pancreatic ductal adenocarcinoma (PDAC). *Methods.* Outcome data for 112 patients of resectable PDAC who received preoperative chemoradiotherapy followed by PD (group I) between January 2004 and April 2010 were retrospectively analyzed and were compared with selected 120 patients who underwent PD alone (group II) in the same period. *Results.* Patients in group I had an incidence of locoregional recurrence of 17.1% compared to 30.8% in group II (*P* = 0.03). There were no statistically significant differences in postoperative morbidity (27.7% versus 30.8%) and mortality (2.67% versus 3.33%). The 1-, 2-, and 3-year survival rates were estimated at 82.1%, 54%, and 28%, respectively, with NCRT and 65.8%, 29.1%, and 10% without (*P* = 0.006). Nevertheless, preoperative chemotherapy did not reduce the 1-, 3-, and 5-year disease-free survival rates, which were estimated at 58%, 36.6%, and 12.5% with NCRT and 51.7%, 18.3%, and 7.5% without (*P* = 0.058). *Conclusions.* The treatment of NCRT followed by PD in patients with PDAC has a significantly lower rate of locoregional recurrence and a longer overall survival than those with surgery alone.

## 1. Introduction

Pancreatic ductal adenocarcinoma (PDAC) is a kind of remarkably highly lethal malignancy, foremost the 5th root cause of loss of life throughout the world [1]. Surgical resection has always been really the only most likely healing alternative. Even so, because of its ambitious tumor expansion as well as recurrence rate [2–4], in addition to the fact that a small section of patients are surgery candidates [5, 6], the actual survival rate of affected individuals is inadequate and simply ranges from 10% to 25.4% [7–10] at 5 years. The unsatisfying benefits of surgical treatment are only able to be enhanced by employing multidisciplinary treatments with adjuvant and neoadjuvant chemoradiotherapy (NCRT).

The additional current publications revealed survival advantages for PDAC patients with the use of adjuvant therapy postoperatively. A meta-analysis has been carried out by Stocken et al. [11] in 2005 from 5 randomized controlled trails, which revealed a 25% diminishment in risk of death in those who obtained chemotherapy and substantial 2 years of survival rates for those who received chemoradiotherapy, in contrast to those who did not (38% versus 25%). Alternatively, up to 30% of individuals had been incapable to complete the course of adjuvant treatment or to receive the designed amount of radiation or chemotherapy typically mainly because of the morbidity and continuous recuperation intervals following surgical treatment [12, 13].

In the contrast, the full course of prescribed chemotherapy is easily completed in NCRT without any delay, and it will presumably enhance effectiveness of chemoradiotherapy.

Even though an extreme variety of phase I/II studies [14, 15] have been published on the potential benefits for NCRT for patients with both resectable and unresectable PDAC, in addition to minimizing the possibilities of local tumor recurrence [16, 17], achieving better local tumor control [17, 18], or tumor downstaging with a subsequent potentially resectable tumor [19–21], unfortunately, no randomized controlled phase III trials comparing NCRT plus surgery versus surgical treatment only have been reported up till now, and as a consequence there are certainly no evidence-based medicine proofs that NCRT can offer any benefits for patients with PDAC. Within the distinction, the entire duration of prescribed chemoradiotherapy is definitely carried out with virtually no holdoff, and it can presumptively improve usefulness for PDAC patients.

Here we reported the principal experience with our large single institution by comparing 5-FU-based NCRT accompanied by PD with surgery alone. It is needed to be realized that NCRT is characterized as any preoperative chemoradiotherapy planning to increase the rate of microscopic tumor clearance and also to reduce the rate of tumor recurrence in this study.

## 2. Materials and Methods

### 2.1. Patients

Between January 2004 and April 2010, 232 consecutive patients with PDAC (limited to TI/T2 TNM staging) who were admitted to the Department of Hepatobiliary Pancreatic Surgery in our institution underwent PD, among whom 112 (48.7%) patients were treated with NCRT preoperatively, whereas the remaining 120 (51.3%) patients underwent PD alone. Patients were included in the study if they were pathologically proven to be PDAC cases postoperatively, and they were excluded if they were not amenable to operation, or if they were other cancer cases or with no cancers.

The approach to NCRT was determined and carried out by individual surgeons. The unique situation in our department was that 1 team of surgeons favored the use of NCRT and 1 team did not. Their choice of treatment was consistent over the period of the study, and this allowed for comparison of treatment between the 2 groups.

Preoperative evaluation of the staging of tumor consisted of physical examination, chest-radiography, abdomen contrast-enhanced computed tomography (CT), magnetic resonance imaging (MRI), and endoscopic retrograde cholangiopancreatography (ERCP). All patients were required to meet the following eligible criteria for tumor resectability: tumors which do not involve major vascular structures including the celiac axis (CA), superior mesenteric artery (SMA), and superior mesenteric/portal vein complex and without extensive peripancreatic lymphadenopathy and/or the absence of distant metastases which were diagnosed radiologically before surgery.

After the completion of chemoradiotherapy, all patients were treated with pancreatic resection for curative intents and underwent no adjuvant chemoradiotherapy postoperatively. The median follow-up time for Group I was 28.6 months (range: 4–70 months) and 24.3 months (range: 9–67 months) for Group II.

### 2.2. Chemoradiotherapy

Chemotherapy was performed as neoadjuvant treatment in 81 of the 112 patients (96.4%). The main agents were 5-FU (600 mg/m^2^, d 1, 8, and 15 for 1 cycle) and gemcitabine (1000 mg/m^2^, d 1, 8, and 15 for 1 cycle). In the study that used only one regimen (*n* = 60), 38 (46.9%) patients were treated using 5-FU, and 35 (43.2%) patients used a gemcitabine-based regimen. Furthermore, gemcitabine and oxaliplatin combinations were used in 8 (9.8%) patients.

Thirty-one of the 112 patients (27.6%) received neoadjuvant radiotherapy (extrabody radiotherapy, EBRT). Doses applied ranged from 46 Gy/23 F to 50 Gy/23 F. No patients received both chemotherapy and radiotherapy.

### 2.3. Operative Finding

After four weeks of chemoradiotherapy, the planned pancreaticoduodenectomy (PD) or partial/total pancreatectomy were performed in all patients for curative intents. In our study, 124 patients (53.5%) underwent a classic PD (Whipple) and 76 (32.7%) underwent a pylorus-preserving PD (PPPD). Partial or total pancreatectomy was performed in 32 (14.8%) patients. R0 resection was achieved in 189 (81.4%) patients, of whom 92 patients were in the NCRT group and 97 patients were in the surgery-alone group. Pathologic specimens were reviewed and staged according to the American Joint Committee on Cancer (AJCC) Guidelines. Pathologic data regarding TNM staging, tumor size, histological differentiation grade, lymph node involvement, lymphovascular invasion, perineural invasion, and surgical margins were recorded.

### 2.4. Followup and Endpoints

All of the included patients were enrolled in our strict follow-up system. After discharge, serum CA-199 and an abdominal ultrasonography (US) and/or contrast-enhanced computed tomography scan was performed approximately 1 month for the initial three months after operation. Thereafter, we screened patients by tumor marker measurement and US every 3 months, and by helical CT every 6 months, and by ERCP or MRI when recurrence was suspected.

The endpoint of this study was time-to-recurrence which was defined as the period between initial pancreatectomy and the diagnosis of recurrence and time-to-death which calculated the duration from the date of transplantation to the date of death for any reasons. All followup data were summarized as of the end of August 2010.

### 2.5. Statistical Analysis

The Chi-square test or the Fisher exact test was used to evaluate the significant differences between the two groups. A proportion of patients with perioperative morbidity and mortality as well as tumor recurrence were compared between the two groups. The Kaplan-Meier curves were constructed for overall survival and disease-free survival, and the log-rank test was applied to compare the survival between the 2 groups of patients. A value of *P* < 0.05 was considered statistically significant.

## 3. Results

### 3.1. Comparison between the Two Groups

Patient demographics, including age, sex, body weight, height, and concurrent illness, were well matched in the two groups (Table 1). The size of the lesion, the histological differentiation, and the depth of tumor invasion in the two groups were also comparable (Table 2). Of the 232 patients, there were 144 (62.1%) males with a median age of 46.2 years (range: 17–67 years) and 88 (37.9%) females with a median age of 38.5 years (range: 24–54 years).

### 3.2. Postoperative Morbidity and Mortality

Data regarding morbidity following neoadjuvant treatment and pancreatic resection were presented for 68 of 232 patients (Table 3). Morbidity included pancreatic fistula, which was defined as all suspect drainage with more than 300 IU/mL amylase-counting for more than 3 days; postoperative intraperitoneal hemorrhage (from arterial or venous vessel, operative field, and gastrointestinal track); lymphorrhea (colorless drainage of more than 300 mL for more than 10 postoperative days); diarrhea (more than three liquid exonerations per day for more than 10 days); delayed gastric emptying, which was calculated by the nasogastric tube (NGT) left in place for 3 days or reinsered because of repeated emesis after removal of the NGT being unable to tolerate a solid diet after the 7th postoperative day; abdominal infection (after the 3rd postoperative day, fever, abdominal distension, and intestinal paralysis appear and last for 24–48 hours, with leukocytosis, hypoproteinemia, and anemia; fluid accumulation is found radiologically); small bowel infraction; pulmonary embolization; atelectasis; and wound infections (Table 4).

The postoperative mortality was calculated as death from any causes within 45 days postoperatively. Postoperative mortality was 2.67% for patients in Group I and 3.33% in Group II. Mortality was not statistically different in the two groups.

### 3.3. Overall Survivals (OS)

**Figure 1:**
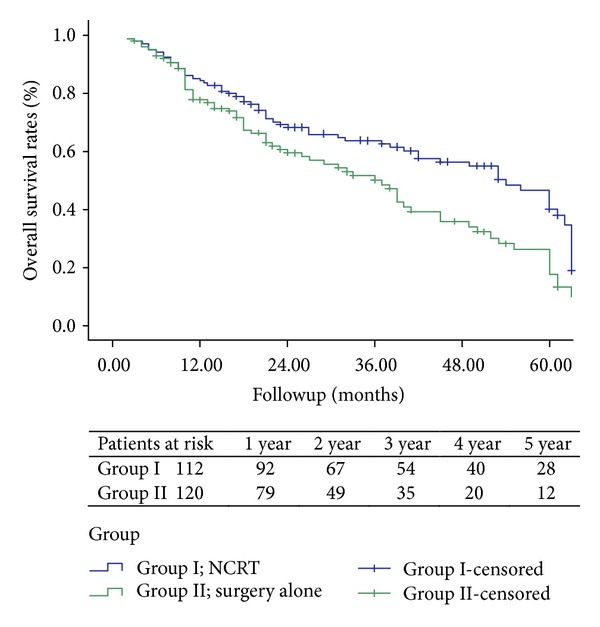
Showing the Kaplan-Meier overall survival curve for the 112 patients receiving NCRT and the 120 patients receiving surgery resection alone. There was no significant difference in overall survival between the two groups (*P* = 0.006).

The analysis of the OS curves between the two groups was revealed in Figure 1, which demonstrated that there was a statistically significant difference between the two groups (*P* = 0.006). The overall survival rates for the 112 patients in NCRT group at 1, 3, and 5 years were 76%, 55%, and 22%, respectively, whereas they were 44%, 25%, and 9% in the surgery-alone group, respectively.

### 3.4. Disease-Free Survivals (DFS)

**Figure 2:**
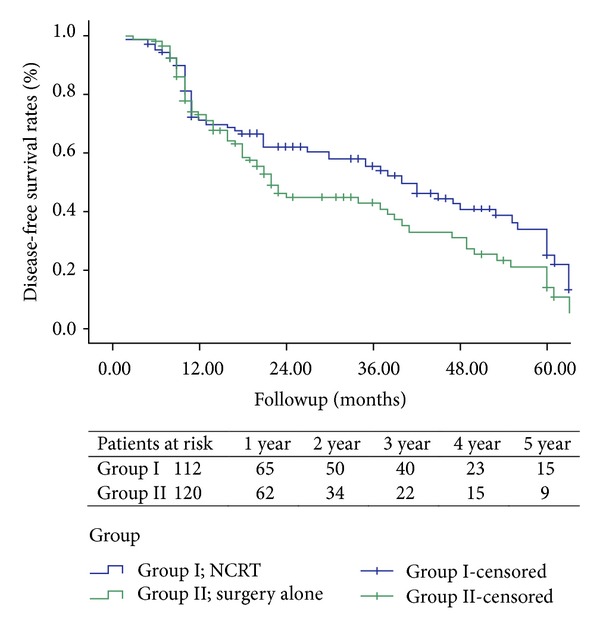
Showing the Kaplan-Meier disease-free survival curve for patients undergoing NCRT (*n* = 112) compared with those patients undergoing surgery resection alone (*n* = 120). There was no statistical difference between the two groups (*P* = 0.058).

The Kaplan-Meier DFS curves of patients between the two groups were compared in Figure 2, which revealed that the DFS was longer in the NCRT group, with the disease-free survival rate of 58% at 1 year, 36.6% at 3 years, and 12.5% at 5 years, and it was 51.7% at 1 year, 22% at 3 years, and 7.5% at 5 years in the surgery-alone group. However, the DFS was not significantly different when the two groups were compared (*P* = 0.058).

### 3.5. Tumor Recurrences

Tumor recurrences were observed in 176 patients. The recurrence rates were 35.3% for the NCRT group and 40.5% for the surgery-alone group, respectively. Focusing on the clinical pathological features of all patients, there were no significant differences between the two groups (*P* < 0.05). Intrahepatic and locoregional lymph nodes metastases were the main first or primary locations of cancer recurrence in both groups (Table 5). Although the overall tumor recurrence rates were not statistically different between the two groups, patients receiving NCRT were more likely to have lower frequency of local lymph node metastasis than patients receiving surgery alone. The frequencies of other locations of postoperative tumor recurrence were similar between the two groups (4.9% versus 5.3%; *P* = 0.02). 

## 4. Discussion

Preoperative chemoradiotherapy is usually a neoadjuvant treatment method. Even though the effective use of adjuvant chemotherapy and radiotherapy in addition to intraoperative radiotherapy (IORT) or EBRT (extrabody radiotherapy) can partially control the local tumor growth and reduce tumor recurrence, there seemed to be tiny impact on the increase of the rate of survival of patients who underwent such procedure, so neoadjuvant therapies are actually suggested by some surgeons, aiming to enhance the resection rate and the 5-year survivals. Theoretically, preoperative chemoradiotherapy has the following advantages when compared with the adjuvant therapy in patients with pancreatic cancer. (1) It can complete the path of adjuvant treatment or obtain the organized quantity of chemotherapy or radiation with no delay virtually [22]; (2) it can downstage tumor classification enabling an improved tumor oncological clearance along with a higher negative surgical margin (R0 resection) [23, 24]; (3) it limits the possible likelihood of cancerous growth seeding due to intraoperative manipulation [25]; (4) it is much more prone to endure it (chemoradiotherapy) prior to surgery; (5) it blocks the oxygen supply to the tumor cells and kills them effectively; and (6) it minimizes the potential risk of pancreatic anastomotic leakage.

NCRT may additionally improve survival after resection for patients with PDAC. Nonetheless, there is actually constrained information concerning the role of NCRT for pancreatic cancer in clinical practice. Preoperative chemotherapy and radiotherapy are nevertheless a place of disputes. Pendurthi et al. [26] retrospectively abbreviated the information of  70 patients who received preoperative and postoperative chemoradiotherapy and found that 27 patients who underwent preoperative chemoradiotherapy were more unlikely to possess lymph node involvement (28% versus 87%, *P* < 0.0006) and a lower rate of positive surgery margins (28% versus 56%, *P* = ns) compared with the 43 patients who obtained chemoradiotherapy after surgery, but there were no significant differences between the two groups in overall survival rates and local tumor control. Similarly, Evans and Pisters [27] from the Department of Anderson Cancer Center confirmed that preoperative chemoradiotherapy did not increase postoperative morbidity, the 3-year survival rate reached 23%, and a less probability of local tumor recurrence was witnessed as a result of a long-term follow-up. In addition, in the Stanford Cancer Center, Joseph Cetal [28] found that preoperative chemoradiotherapy was tolerated in locally advanced tumors without having to incorporate the operation risk and downstage the tumor TNM stage, as well as to increase the oncological clearance rate.

The role of preoperative chemoradiotherapy in extended survival for patients with pancreatic cancer remains not noticeable currently, but NCRT has been considered as one of the most reliable treatment methods within the treatments for individuals with locally advanced pancreatic cancer.

The parameters used to classify the pancreatic cancer into resectable category are typically depending on primary tumor TNM stage, lymph nodes status, and adjacent major organs conditions. The surgical resection margin status and the existence of lymph nodes metastases were found to be the most significant determinants of survival after surgery [29, 30]. In our study, patients who received pancreatectomy alone were more prone to have local lymph node metastasis (*P* < 0.001). Overall survival was statistically different for those who received neoadjuvant therapy when compared with those who received surgery alone (*P* = 0.969), even though there was no statistical difference in disease-free survivals among the two groups.

Our study can be criticized for its patient selection and lack of pathological diagnosis preoperatively, as well as the retrospective nature of assessment of outcome. Nonetheless, considering the existing debate in the application of NCRT for PDAC, it highlights several significant things for surgeons. First, it is very important to determine which patients are more or less likely to benefit from the use of  NCRT. Second, the preoperative criteria and definitions for resectability and unresectability are clearly keys and have to be standardized, and the necessity for a histological diagnosis is additionally an essential point for the patients who are initially unresectable. Third, it is also considerable to distinguish between the prognostically favorable groups of patients with intrapancreatic bile duct cancer from individuals with PADC due to the effects of the neoadjuvant treatment on histology of the primary tumor. Additionally, the survival rates reported in our research also compare and contrast positively towards the survival data of other retrospective neoadjuvant treatment series.

## 5. Conclusion

The frequently acknowledged conventional strategy to affected individuals with resectable pancreatic tumors is pancreaticoduodenectomy accompanied by 5-FU-based chemotherapy or radiotherapy. In the absence of randomized controlled trails, the application of neoadjuvant therapy for resectable pancreatic cancer remains disputable. For marginally unresectable tumors, neoadjuvant chemoradiotherapy may turn out to be a highly effective strategy for determining which patients might possibly benefit from surgical exploration and experimented with resection.

## Figures and Tables

**Table 1:** Clinical characteristics of the patients.

Demographics	Group I	Group II	*P* values
Sex (male/female)	75/37	69/51	0.773
Age (year)	45.9 ± 9.8	45.5 ± 9.3	0.672
Heights (cm)	166.5 ± 6.1	165.2 ± 5.8	0.914
Body weight (kg/m^2^)	57.6 ± 10.3	59.9 ± 7.6	0.254
Concurrent illness			
Hypertension	21	24	.ns*
Pulmonary tuberculosis	4	3	.ns
Diabetes mellitus	13	10	.ns
COPD*	11	14	.ns
Cholelithiasis	16	22	.ns
GERD*	5	7	.ns
Endometriosis	4	4	.ns
Others	13	16	.ns

*COPD: chronic obstructive pulmonary diseases; *GERD: Gastroesophageal reflux disease; ns: not significant.

**Table 2:** Operative and pathological characteristics of both groups.

Characteristics	Group I	Group II	*P *values
Tumor location (head/body/tail)	98/14	96/24	.ns
Tumor size (mm)	3.2 ± 1.3	3.5 ± 0.8	.ns
Serum CA199 (U/mL)	210.7 ± 45.6	284.3 ± 55.7	.ns
Types of surgery			
Whipple*	65	59	.ns
PPPD*	35	41	.ns
Partial or total pancreatectomy	12	20	.ns
Pathological differentiation (well/moderate/poor/others)	12/74/23/3	14/76/25/5	.ns
TNM* staging (I/II/III-IV)	58/49/5	49/64/7	.ns
Surgery margins (R0/R1/R2)	95/17/0	96/24/0	.ns
Operative time (min)	615 ± 180	635 ± 210	.ns
Blood loss (mL)	1120 ± 350	1240 ± 430	.ns
Hospital stay (day)	11.5 ± 4.3	12.3 ± 3.5	.ns

*Whipple: standard pancreatoduodenectomy. PPPD: pylorus-preserving pancreatic resection. T: tumor. N: lymph nodes. M: metastasis.

**Table 3:** Postoperative mortality and morbidity between the two groups.

Objects	All patients	Group I	Group II	*P* values
Morbidity	29.3%	27.7%	30.8%	0.123
Mortality	3.02%	2.67%	3.33%	0.123

**Table 4:** Number of complications between the two groups.

Complications	Group I	Group II
Pancreatic fistula	7	9
Intraperitoneal hemorrhage	3	4
Lymphorrhea	5	6
Small bowel infarction	2	2
Diarrhea	4	5
Pulmonary embolization	2	1
Atelectasis	3	2
Delayed gastric emptying	5	8

Total	31	37

**Table 5:** Comparison between the two groups regarding the first location of tumor recurrences.

Metastasis site	Group I (no. and %)	Group II (no. and %)	*P* values
Intrahepatic	35 (42.7%)	31 (33.1%)	.ns
Locoregional	14 (17.1%)	29 (30.8%)	0.032
Peritoneal	12 (14.8%)	11 (11.7%)	.ns
Pulmonary	10 (12.2%)	12 (12.9%)	.ns
Retroperitoneal	7 (8.5%)	6 (6.4%)	.ns
Others	4 (4.9%)	5 (5.3%)	.ns
